# Courtship at reaching-range in the túngara frog

**DOI:** 10.1007/s00359-026-01830-4

**Published:** 2026-08-10

**Authors:** Olivia R. Chung, Marcos Gridi-Papp

**Affiliations:** Department of Biological Sciences, University of the Pacific, Stockton, CA 95211 USA

**Keywords:** Breeding, Communication, Amphibia, Calling, Amplexus, Advertisement call

## Abstract

Courtship in frogs can mediate the final approach between the sexes before amplexus and spawning. It can involve male and female calling, touching, and guidance to the spawning site. Courtship behaviors have been described for several species in nature but little experimentation has been done to uncover the biological mechanisms that control them. We characterized the close-range courtship (within 10 cm) interactions in the túngara frog, an emerging model organism, to provide a quantitative baseline in a neutral arena in support of future experiments. In this initial assessment, we isolated each pair to examine only the social stimulation that is intrinsic to the pair. Among 81 pairs, 80% entered amplexus and of those 57% laid eggs. Males called before amplexus in 86% of the trials in spite of the female’s proximity. The female was inferred to execute the final approach before amplexus in 79% of the events. Egg laying was concentrated in the period 6–8 h after sunset and it could be followed by calling. Multiple bouts of egg nest building were observed in 1/4 of the trials and physical contact other than amplexus was common. Male strategy, the last approach before amplexus, and egg laying were not influenced by age or body size but females with high body condition were more likely to lay eggs. Our data revealed that courtship in the túngara frog is not necessarily terminated when the male is approached by the female, but additional close-range interactions may mediate the onset of amplexus and reproductive success.

**Supplementary Information:**

The online version contains supplementary material available at https://doi.org/10.1007/s00359-026-01830-4.

## Introduction

Advertisement and courtship precede reproduction in many animals. Calling is the main mode of advertisement in frogs but they can also employ visual (Lindquist and Hetherington 1998; Hödl and Amézquita 2001; Hartmann et al. 2005), seismic (Lewis and Narins 1985; Cardoso and Heyer 1995), and chemical (Still et al. 2019) signaling. The diversity of frog calls has been characterized in detail and experimental studies have demonstrated the effect of calls in female attraction, species recognition and mate choice (Littlejohn 1965; Ryan 1980; Gerhardt 1994; Toledo et al. 2015). In addition to attracting females, advertisement calls also ritualize conflicts with other males in the maintenance of territories, minimum separation distance, and other aggressive interactions (Klump and Gerhardt 1992; Halliday and Tejedo 1995; Bee and Perrill 1996). Advertisement calling has been shown to be influenced by body size and condition, in addition to the physical and social environments in the chorus (Gerhardt and Huber 2002; Wells 2007).

Courtship can follow advertisement once the male detects the presence of a female (Wells 1977). A wide diversity of courtship behaviors has been described in frogs, involving calling by the male, duetting with the female and ritualized movements by either sex (Townsend and Stewart 1986; Tobias et al. 1998; Bosch and Márquez 2001; Haddad et al. 2005; Toledo et al. 2015). The male may produce calls that are specific to courtship or he may continue emitting advertisement calls but usually at a higher rate. Signaling during courtship has been described as more vigorous than during advertisement, possibly as an attempt of the male to consolidate his election by the female. Another (non-exclusive) possibility is that calling may accelerate ovulation and prepare the female for egg laying (Lea et al. 2001). Male calls have been found to increase circulating sex hormone levels in female túngara frogs (Lynch and Wilczynski 2006) and stimulate follicular development in the ovaries of *Alytes* (Lea et al. 2001). In a previous study (unpublished) we showed that stimulation by playback of advertisement calls sufficed to cause isolated female túngara frogs (without a male) to oviposit. Vigorous calling during courtship may add to the stimulation provided by the chorus and prepare the female to lay eggs. Finally, another role of courtship in some species is the physical guidance of the female by the male to the site of egg laying (Townsend and Stewart 1986; Haddad et al. 2005; Lucas et al. 2008; Oliveira Filho and Giaretta 2008).

While courtship interactions have been described in several species, the influence of intrinsic factors such as body size and condition or extrinsic factors such as social stimuli on each of the mates and their breeding success remains mostly unknown. We initiated an experimental investigation of the close-range interactions in frogs with the goal of identifying the main driving factors of courtship behavior. Like advertisement calling, courtship interactions may also be responsive to a wide range of factors intrinsic or extrinsic to the individual.

We studied the túngara frog, *Engystomops (= Physalaemus) pustulosus*, a model species for reproductive behavior in anurans (Ryan 1985). It is a tropical species that can breed most months of the year, depending on precipitation, with males forming choruses at ponds, ditches and puddles. These leks have highly male-biased sex ratios with a few large males taking most of the matings (Ryan 1983). Males only call when floating, most frequently from the edge of a pond. Their calls attract gravid females and this attraction is increased when the calls are suffixed with optional notes (chucks) that increase call complexity (Ryan 1980). Calling imposes energetic costs and its ornamentation with added complexity increases predation risk to the individual (Tuttle and Ryan 1981; Bucher et al. 1982; Bernal et al. 2007). Males do not engage in scramble competition and do not actively search for females (Ryan 1983). They remain relatively stationary while calling, and switch from calling to clasping once the female has approached within a few centimeters. Within this reaching range, however, the male will also indiscriminately clasp other males and heterospecifics. A female typically listens to multiple males from at least 10–20 cm distance before approaching the chosen mate (Ryan 1983; Akre and Ryan 2011).

We used a captive colony of túngara frogs to monitor courtship with a reliable source of breeding events under controlled conditions. Breeding in this colony takes place throughout the year without requiring hormones or other chemical treatment. Adults are given a monthly breeding opportunity in which each pair is placed for a night in a small enclosure with water and this consistently results in about half of the pairs laying eggs. We used infrared cameras to examine the interactions within a pair when the animals were within reach of each other and the female was likely gravid (had mature eggs) but had not yet been stimulated with calls.

The goal of this study was to provide a quantitative characterization of courtship in an emerging model organism: the túngara frog. In a “bottom-up” approach to the complexity of courtship behavior, we started with a neutral arena in which the mating pair was deprived of any external social stimulation, such as chorus sounds. While unnatural in a chorusing species, this setting exposed the intrinsic interactions within a pair and allowed us to explore their relationships with size and age. This provided a “baseline” for the potential effects of social stimulation which should be addressed in a follow-up study.

## Materials and methods

### Animals and husbandry

A colony of túngara frogs containing ~ 144 breeding pairs was used in this study. Its breeding and culling protocols are designed to minimize the loss of genetic variability and to maintain a stable population structure. Each individual is identified and its biometrics and breeding history are tracked. Since the inception of the colony in 2013 it has occasionally incorporated new individuals donated by collaborators working in Panama and no signs of inbreeding depression have been detected. The subjects of this study were healthy, breeding adults between 230 and 830 days in age, except for an outlier male that was 1072 days old. He was not an outlier in body length or body mass, his behavior did not stand out and he produced a regular egg nest so we maintained his results in the analysis. All the analyses produced the same conclusions when ran without his data. This study followed IACUC protocol 22R07 of the University of the Pacific.

The frogs are fed wingless fruit flies (*Drosophila melanogaster*) dusted with a supplement (Reptivite+D3, Zoo-Med) on alternate days. The colony is maintained in a 12 h daylight cycle. Room temperature is cycled daily between 27° C and 25° C. Frogs are housed in 38 L glass tanks each containing six males and six females. Peat moss and coconut coir form the bedding and four black 59 ml portion cups are added to each tank up side down as shelters. Misting is provided in alternate days (0–750 ml/tank) and the excess drains out through an orifice at the bottom of each tank. The bedding is replaced and the tanks are bleached monthly. Deionized water reconstituted with minerals is used in every aspect of husbandry.

Males and females are housed together, but without a pool of water, there is no calling or egg laying in the tanks. We control reproduction by moving the frogs in pairs into breeding enclosures with water where they can call and spawn. The identity of the parents of every new brood is known. In the field, túngara frogs are known to breed when it rains at any time of the year (Ryan 1985; Marsh 2000). Females can lay eggs multiple times in the year and mark-and-recapture data indicate that some females can return to be pond to breed after approximately one month (Ryan 1985). We mimic this pattern in our lab and maintain an interval of at least a month between breeding opportunities for each female.

### Courtship experiments

Each trial consisted of a male and a female placed in physical and acoustic isolation from any other frogs into a tested breeding enclosure in which courtship and spawning behaviors were monitored. The female and the male were initially weighted and identified through a picture of the highly variable pigmentation pattern of their ventral skin. The female was released into the breeding enclosure while the male was added to it within a closed 50 ml test tube with breathing holes. This tube was humidified but did not contain a pool of water so the male was prevented from calling or clasping the female until all pairs (6 per day) were prepared and positioned under their cameras.

Each breeding enclosure was a 9.7 × 9.7 × 12.4 cm (internal length x width x height) acrylic display case with a meshed lid. It received 170 ml of water which produced a 1.8 cm depth. A 10.5 × 3.2 × 0.3 cm acrylic plate was inserted touching one wall at its bottom and the opposite wall at a height of 4.5 cm. This formed a ramp that the animals could climb on to stay dry or immersed at any depth up to 1.8 cm. The meshed lid of the enclosure was fitted with an infrared-sensitive camera having infrared LEDs (Arducam B0506) that allowed for video monitoring in the dark. This should not have been disruptive to the animals because túngara frogs have not shown sensitivity to infrared light (Cummings et al. 2008). The breeding enclosure was placed in a custom sound-isolation chamber having 9 layers of plywood, cellulose, and mass loaded vinyl that prevented calls produced in one chamber from being heard in a neighboring chamber (unpublished). The transmission loss between the sound-isolation chambers was 51 ± 4.6 dB (mean ± SD) at 800 Hz and higher levels of amplitude loss were produced at higher frequencies.

Once all pairs were positioned and the cameras were activated, the male of each pair was released from the test tube to meet the female in the breeding enclosure and the test tube was removed. The movements of both animals were monitored continuously throughout the trial. Images 1985 × 1140 pixels in size were recorded to monitor the bottom and walls of the breeding enclosures at a rate of 1 frame per minute. Each pair was monitored for a period of 24 to 72 h. After each trial, the body masses of the adults were measured and they were returned to the colony. Time-lapse video recordings were generated from the images and scored to analyze courtship interactions based on each individual’s position, and the presence of a foam egg nest. Male calling behavior was inferred from the characteristic floating posture with the lungs inflated and the vocal sac at various degrees of inflation. In nature, males typically call for hours and are not seen floating inflated without ever calling so both false-positives and false-negatives seemed unlikely in our image-based inferences. Faulty equipment prevented us from obtaining audio recordings in this experiment but we validated our inferences in a subsequent experiment with microphone and camera recordings. Blind analysis of the video and audio recordings revealed that out of 25 trials, video scoring correctly identified calling in all pairs, without producing false-positives or missing any real calling bouts.

We also used infrared imaging to infer which individual approached the other immediately prior to amplexus. While our frame rate was coarse (1 frame/min) the frogs spent most of the time still or stationary and exhibited occasional small movements which showed continuity in subsequent images. We assigned the “last approach” to the individual who moved the greatest distance from its position in the last frame (1 min) prior to amplexus to its position in the first frame of the pair in amplexus. This is an inference as additional movement could have occurred between pictures. When measured over multiple trials, however, it may provide a useful indication of which sex is more likely to close the final distance before amplexus. It only refers to movement, however, and does not incorporate any signals that the animals might emit to trigger amplexus.

Stress levels are of real concern both in the husbandry of the colony and in any behavioral experimentation involving animals. The experiment described in this report is almost identical in procedure and materials to that of the monthly breeding opportunity that we provide to our animals within the regular husbandry of the colony. This procedure has been validated for more than a decade and while it involves handling the animals for the identification photographs and weighing, about half of the pairs lay eggs in their first night together. This proportion is high if one considers that in the field, only females that are ready to lay eggs approach the pond (Ryan 1985), whereas in our colony we include all adult individuals without being sure of their reproductive readiness. Our experiment differed from regular husbandry, however, in keeping the male separated inside a perforated test tube for about 20 min before commencing the trial. This manipulation appeared to be well-tolerated by the males as our cameras showed that most of them engaged in exploration of the enclosure and calling a few minutes after being released.

### Data analysis

Binomial tests were used to test if the proportions of males that clasped the female with or without prior calling were different from 0.5 and to test if the proportion of amplexuses in which the last approach was executed by each sex differed from 0.5. A Chi-square test was used to test if the proportion of pairs that laid eggs was different between pairs in which the male called or did not call prior to amplexus. Logistic regression analyses were conducted to evaluate the predicting ability of snout-to-vent length (SVL), body mass, body mass index (BMI = body mass / SVL^2^), age of male or female and calling preceding amplexus, on binary behavioral outcomes, namely calling, amplexus, sex of the animal that executed the last approach before amplexus, and success at egg laying. With multiple independent variables, we employed a stepwise forward variable selection method based on the significance of the score statistic for variable entry and the probability of a likelihood-ratio statistic for variable removal.

## Results

In 81 breeding trials, the male clasped the female in 65 of the pairs and more than half (37) of them produced egg nests. This matches the breeding success normally observed in our husbandry and indicates that the experimental manipulation did not stress the animals to the point of distorting the results. Only four males neither called nor clasped the female and therefore did not contribute data to the analyses of reproductive behaviors.

**Fig. 1:**
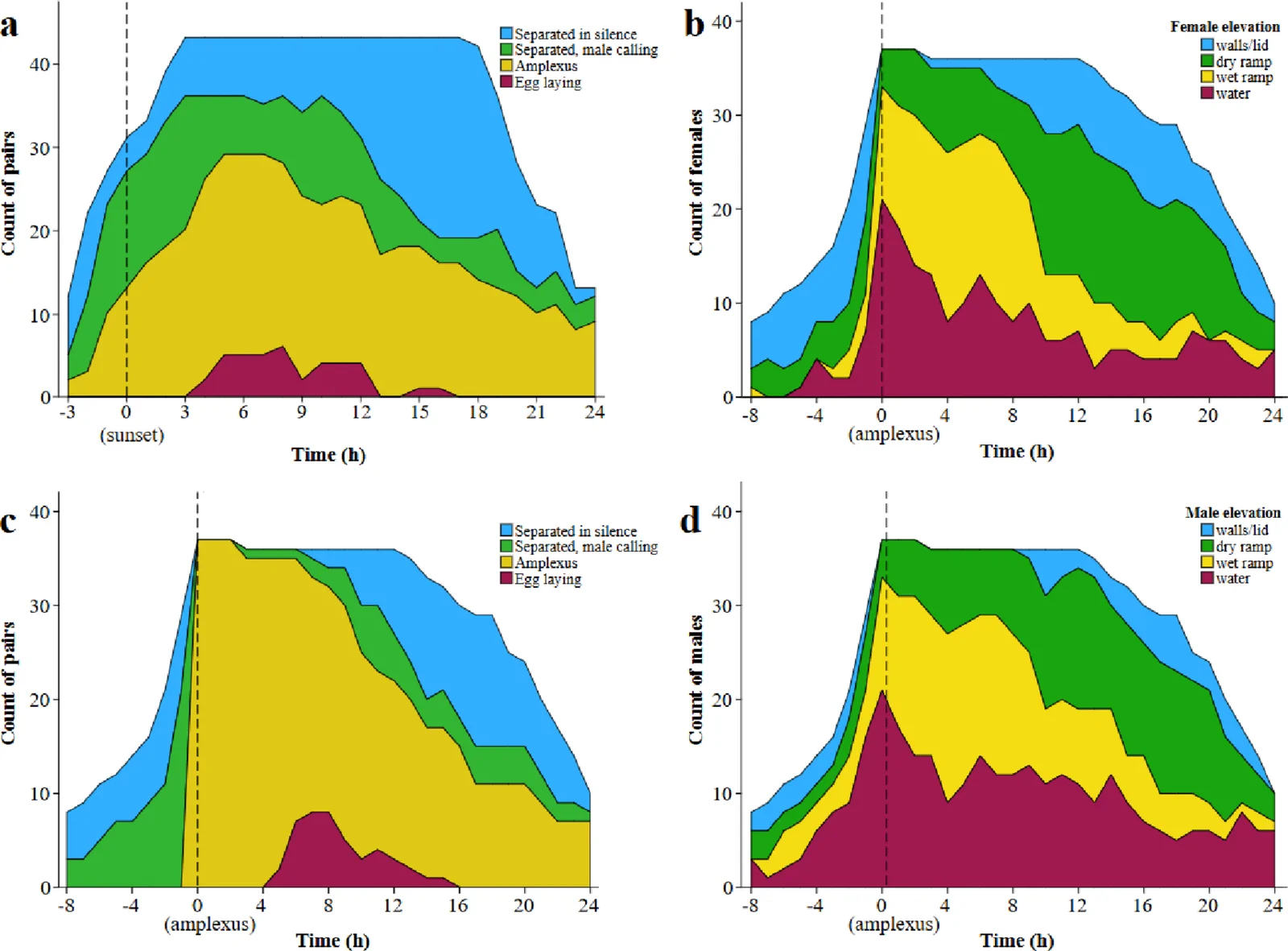
Timing of close range interactions within the breeding pair. **a** Number of pairs at various breeding stages by hour in relation to the artificial sunset of the colony (set to 2 pm). **b** Number of females at various elevations by hour in relation to the start of amplexus. Females mostly stayed dry when not in amplexus or laying eggs. **c** Number of pairs at various breeding stages by hour in relation to the start of amplexus. **d** Number of males at various elevations by hour in relation to the start of amplexus

**Fig. 2:**
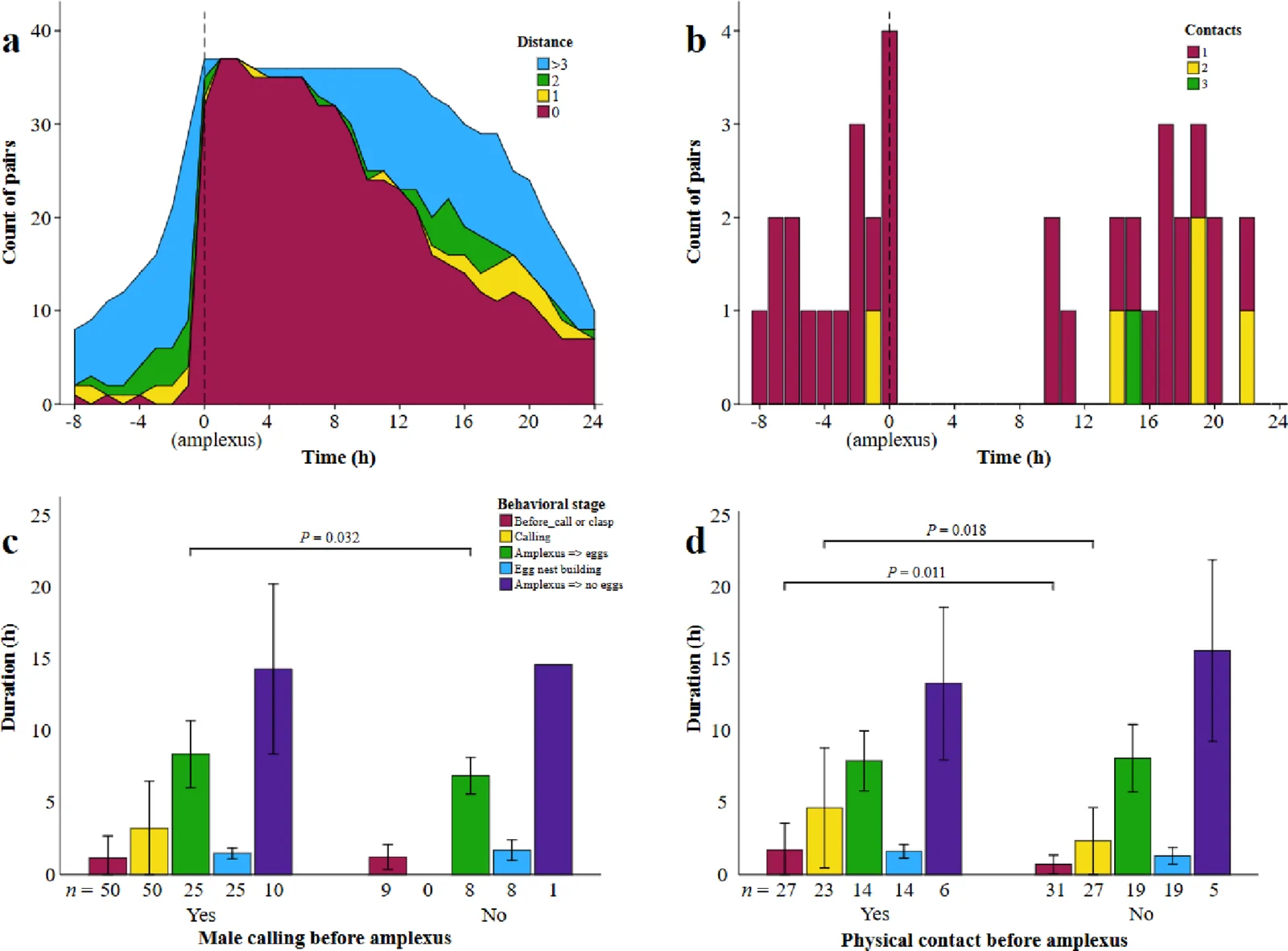
Timing of close range interactions within the breeding pair. **a** Number of pairs at various horizontal distances between individuals (body lengths) by hour in relation to the start of amplexus. **b** Number of pairs with 1, 2 or 3 non-amplexus contacts by hour in relation to the start of amplexus. **c** Duration of behavioral stages in trials grouped by presence or absence of male calling prior to amplexus. Legend: Before call or clasp = time between the start of the trial and the start of either male calling or amplexus; Calling = duration of male calling before amplexus; Egg nest building = time from when the first reproductive secretions became visible to when the amplexus was terminated or the pair moved away from the egg mass. Amplexus = > eggs = duration of amplexus that was followed by egg nest building; Amplexus = > no eggs = duration of amplexus that was terminated by the pair without egg nest building. **d** Duration of behavioral stages in trials grouped by presence or absence of physical contact between the mates prior to amplexus. Error bars = standard deviation

Calling and amplexus started hours before the artificial sunset (set for 2 pm), possibly because the breeding enclosures were dark during the entire experiment (Fig. 1a). Egg nest building started 3 h after sunset and continued throughout the night with most activity from 6 to 8 h after the sunset. Amplexus lasted anywhere from 4 h to the remaining duration of the experiment, and most breeding occurred 6 to 8 h after the initiation of amplexus (Fig. 1c). Calling happened both before and after amplexus, however the proportion of males calling was larger before amplexus (Fig. 1c). While males spent most of their time in the water, females remained in the water when in amplexus but would otherwise spend most of the time in the dry parts of the enclosure before and after amplexus (Fig. 1bd). The distance between the female and the male was variable when not in amplexus with greater distances appearing more common at later times (Fig. 2a). Interactions involving physical contact that did not immediately lead to amplexus were observed in most pairs and occurred before and after amplexus, with most occurrences shortly before amplexus (Fig. 2b).

Calling lasted 3.4 ± 3.5 h (mean ± SD; *n* = 50). Pairs that built egg nests preceded it with 8.0 ± 2.2 h (*n* = 33) of amplexus. Some pairs did not lay eggs and terminated their amplexuses after 14.3 h ± 5.6 (*n* = 11) or were interrupted at the end of the trial. Egg nest building took 1.5 ± 0.5 h (*n* = 33). The durations of most of these behavioral stages were similar between trials in which the male called or did not call before amplexus (Fig. 2C). Amplexus before egg laying, however, was longer (*t* = 2.3, *P* = 0.032) when it was preceded by male calling (8.4 ± 2.3 h; *n* = 25) than when amplexus was not preceded by calling (6.9 ± 1.3 h; *n* = 8). Males took longer to start calling (*t* = 2.7, *P* = 0.011) in trials with amplexus preceded by non-amplexus contact between the mates (1.7 ± 1.9 h; *n* = 27) than in trials without such contacts (0.7 ± 0.7 h; *n* = 31). In addition, males called for longer (*t* = 2.5, *P* = 0.018) in trials with non-amplexus contact before mating (4.7 ± 4.2 h; *n* = 23) than in trials that lacked these interactions (2.3 ± 2.3 h; *n* = 27).

**Fig. 3:**
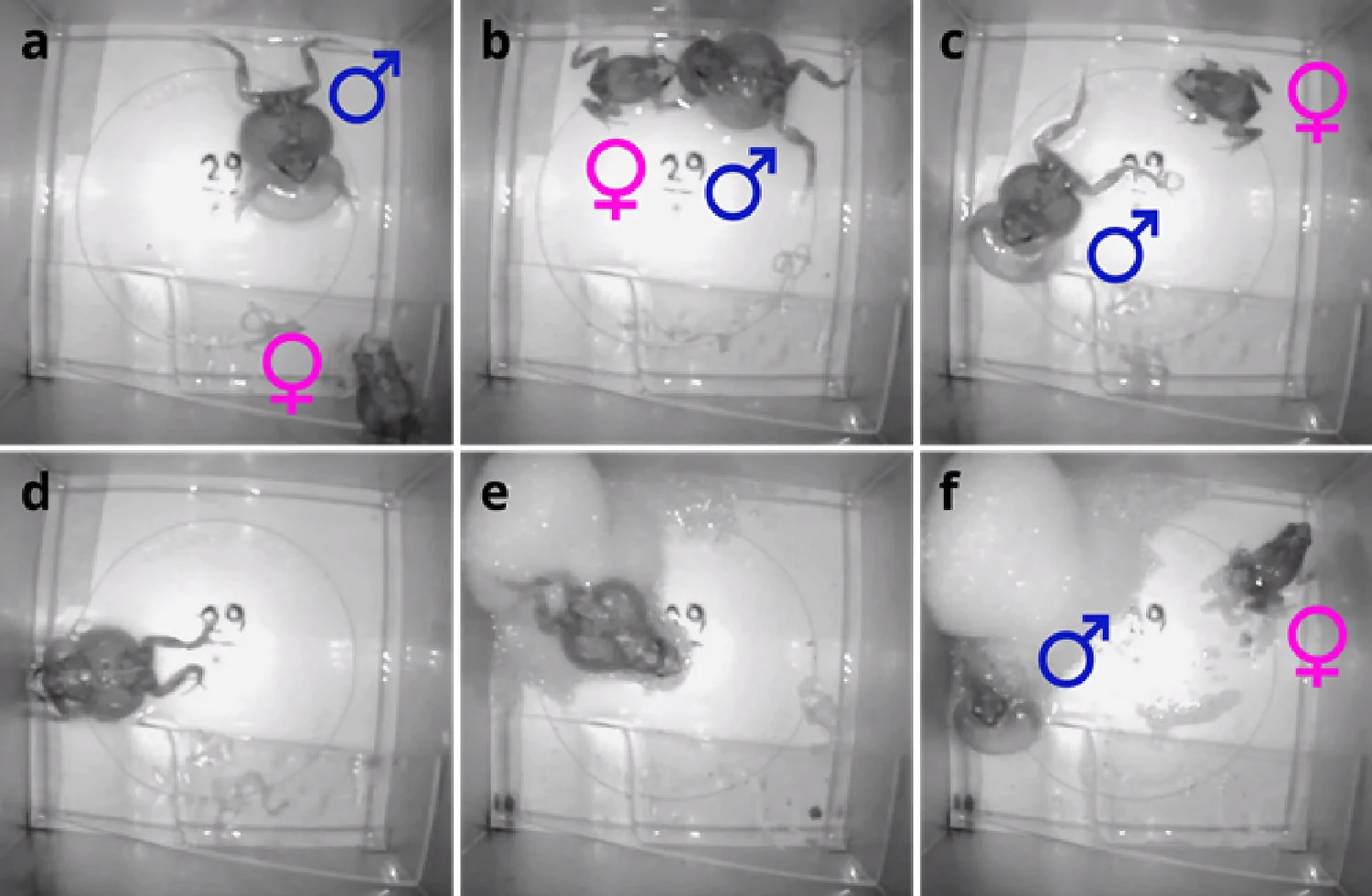
Typical courtship during breeding trials. **a** Female on the ramp listening to the calls of the male in the water. **b** The female enters the water and performs non-amplexus physical contact with the male between calls (vocal sac partially deflated). **c** Last frame before amplexus. **d** Pair in amplexus at the position previously occupied by the male (rated as last approach by the female because she showed the greatest displacement since the previous frame). **e** Construction of the foam egg nest (the pair remains in amplexus). **f** Egg nest completed, amplexus terminated, and the male is back to calling

**Fig. 4:**
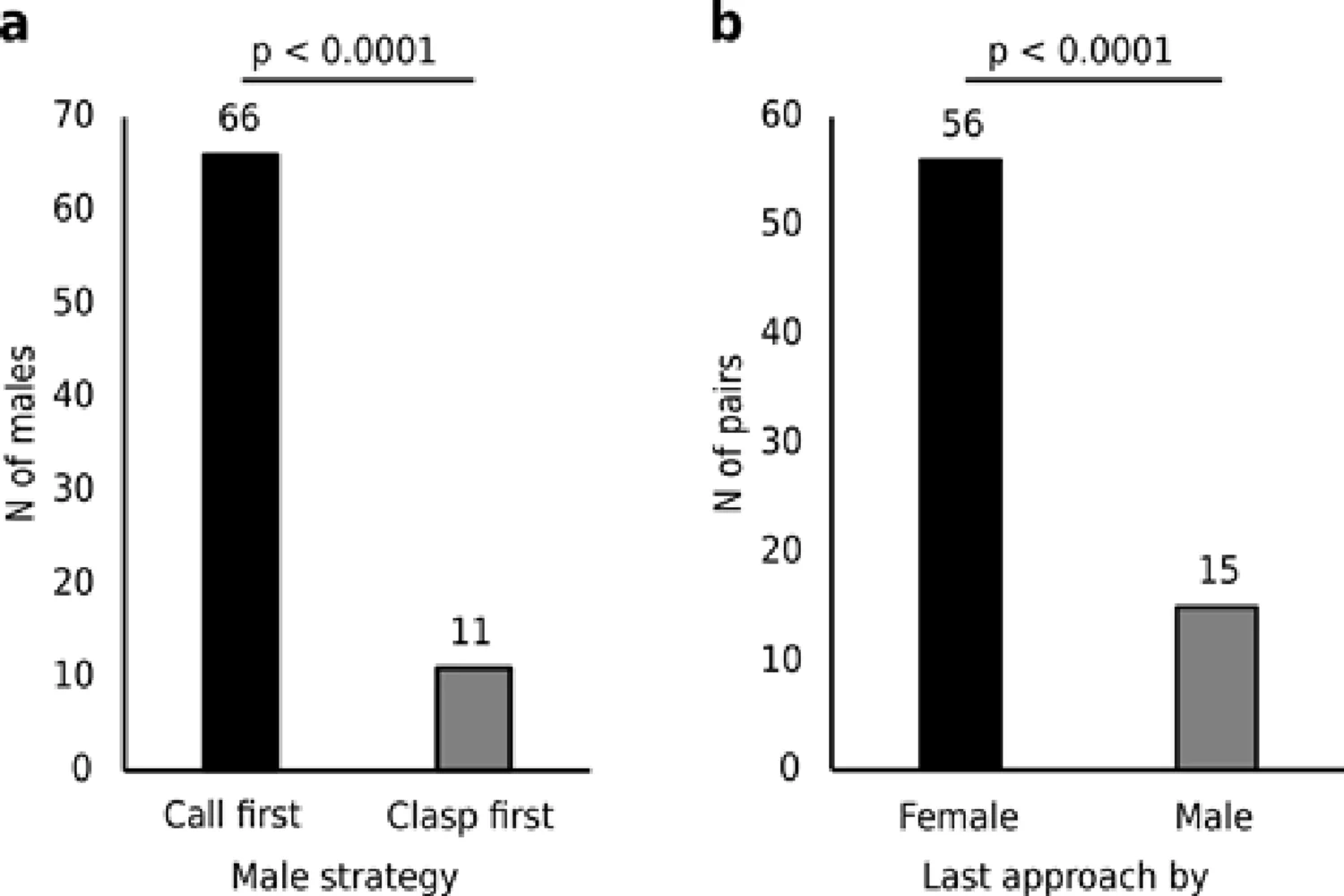
Courtship behavior at reaching range. **a** Number of males that initially called or clasped the female during breeding trials. The majority started with calling instead of clasping. **b** Number of pairs in which the last approach before amplexus was executed by the female or by the male during breeding trials. Females executed the last approach preceding most amplexuses. The number of cases is indicated above each bar

In a typical trial, the female positioned herself at the top of the ramp (Fig. 3a) or higher against a corner of the enclosure (Supplementary Materials S1_video_24h.mp4 ). The male usually went into the water and inflated himself to call (Fig. 3a-c) or briefly sat on the ramp before going into the water to call. More males (66) called before amplexus than clasped the female without calling (11; binomial test, *P* < 0.0001, *n* = 77; Fig. 4a).

The female could move freely around the male without being clasped and the pair engaged in various forms of physical contact that was not part of amplexus (Fig. 3b). The female would frequently approach and contact the calling male or the male could contact the female on the ramp between bouts of calling. While the roles of these interactions could not be characterized from our observations, their occurrence throughout the trials indicates an unexpected level of elaboration in the closest phase of courtship. In one case, the male remained mostly on the dry portion of the ramp instead of entering the water to call. The female moved around the enclosure and was imaged at 4 times next to the male with one or both of the palms of her forelegs on his back (Supplementary Materials Fig S2). He eventually clasped her on the following night but they later terminated the amplexus without building an egg nest.

During male calling, the female approached him by moving down the ramp slowly toward the edge of the water or by jumping into the water depending on the location of the male relative to the ramp (Fig. 3c). When the male was calling from the bottom of the ramp (wet ramp), the female could approach him directly while facing him (Fig. 3c) or she could approach him moving backwards (Supplementary Materials S3_video_retrograde.mp4). The final approach (last 1 min) before amplexus was executed by the female more frequently than by the male (binomial test, *P* < 0.0001 *n* = 71; Fig. 4b).

**Fig. 5:**
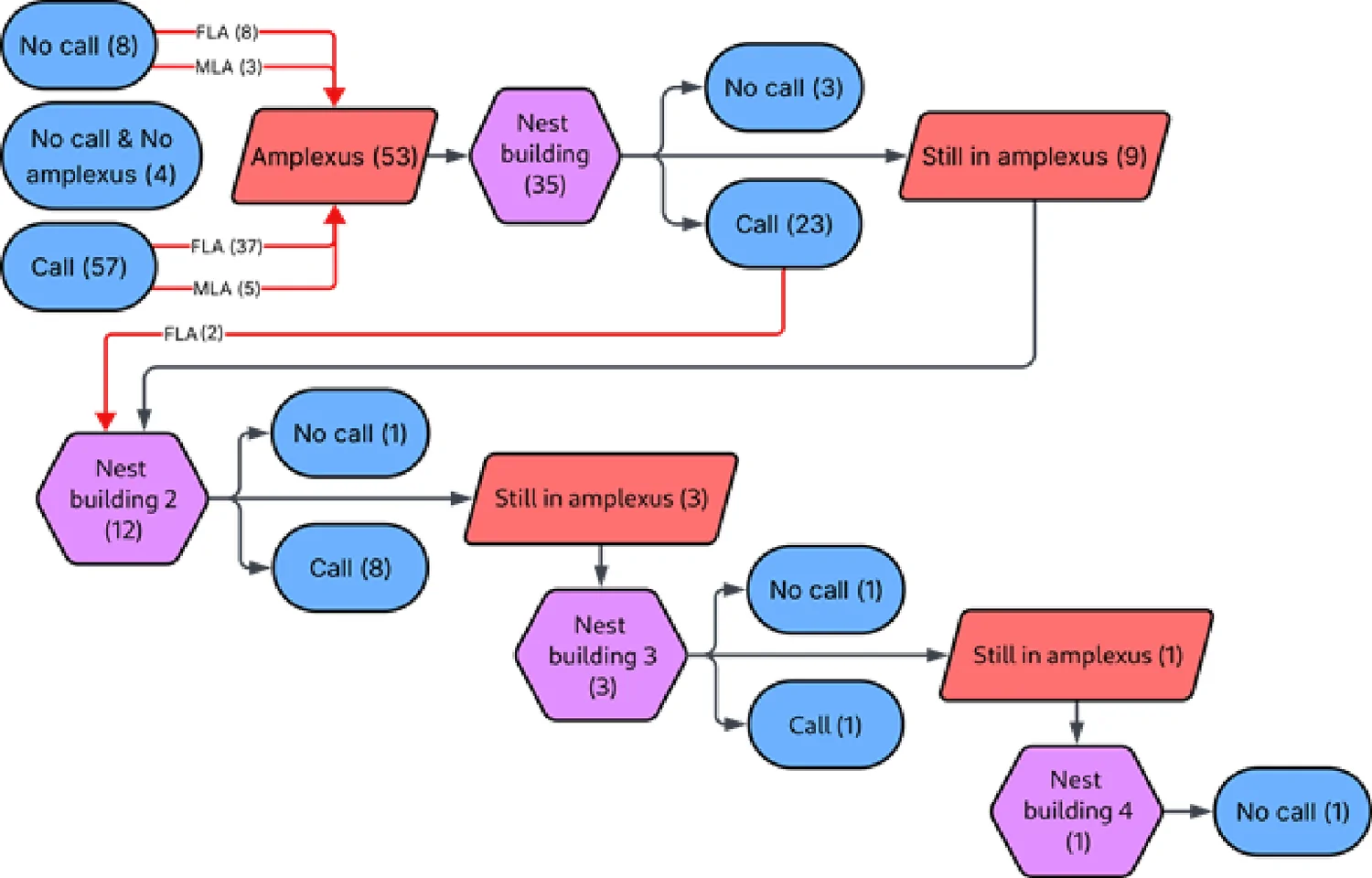
Sequence of behaviors in all trials (*n* = 69) except the ones that terminated amplexus before egg nest building (*n* = 12). FLA = Female last approach. MLA = Male last approach. The arrow split after nest building indicates that some pairs will remain in amplexus while others will split and the male may resume calling or stay silent for the rest of the trial. Numbers of cases are indicated within parentheses

**Fig. 6:**
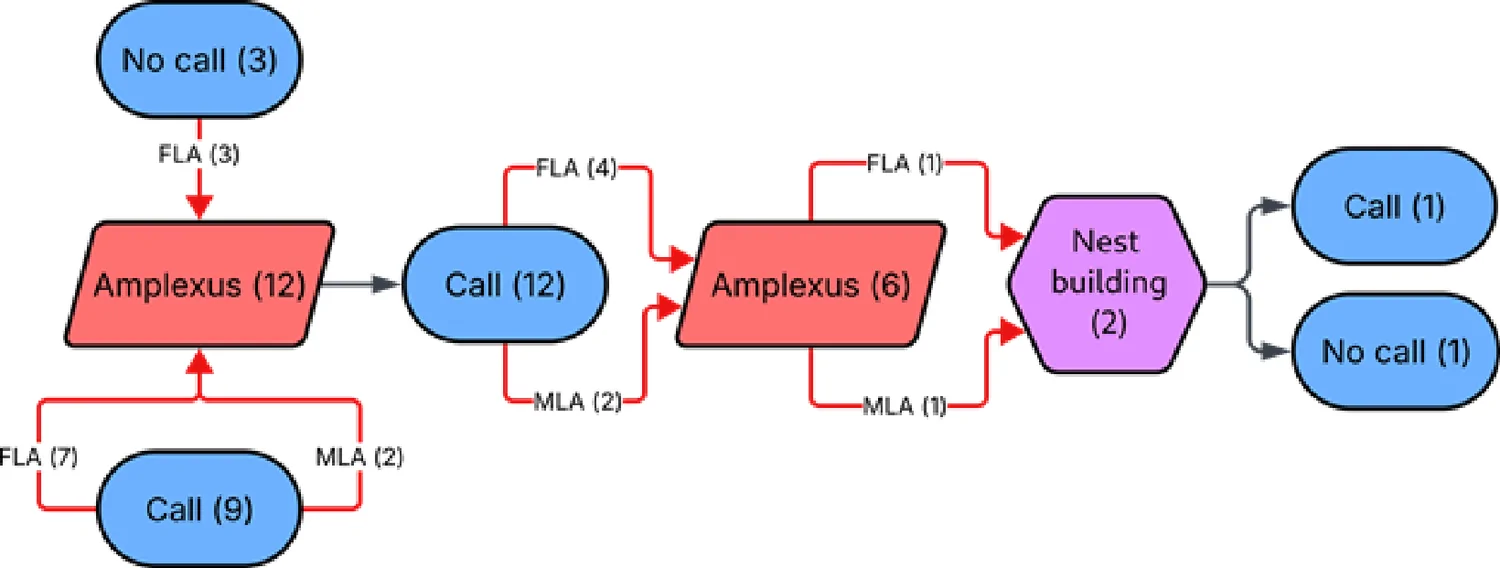
Sequence of behaviors within reaching-range courtship in pairs that terminated amplexus before egg laying (*n* = 12). FLA = Female last approach. MLA = Male last approach. Numbers of cases indicated within parentheses

In 77 breeding trials, amplexus occurred at least once in 65 pairs. Amplexus persisted until egg nest building (Figs. 3e and 5) in the majority of the trials and the male frequently resumed calling within the same night (Figs. 3f and 5). Multiple amplexuses were observed in several pairs that succeeded at building egg nests in the first amplexus. The female executed the last approach when the male resumed calling in all of these cases, independent of the addition of an egg mass to the nest. In 18 trials, amplexus lasted until the end of the trial (~ 24 h) without nest building. In 12 cases, amplexus was terminated during the trial prior to egg nest building (Fig. 6). Of these 12 cases, 6 pairs ended the amplexus, the male resumed calling and after a while the male clasped the female a second time. Only two of the pairs that experienced recurring amplexus built an egg nest on the second attempt. Our data do not provide a clear indication of the cause of the amplexus events that failed to produce eggs. In most cases, the female executed the last approach and the male resumed calling after amplexus was terminated. Both sexes had therefore exhibited behaviors that indicate mating readiness.

**Fig. 7:**
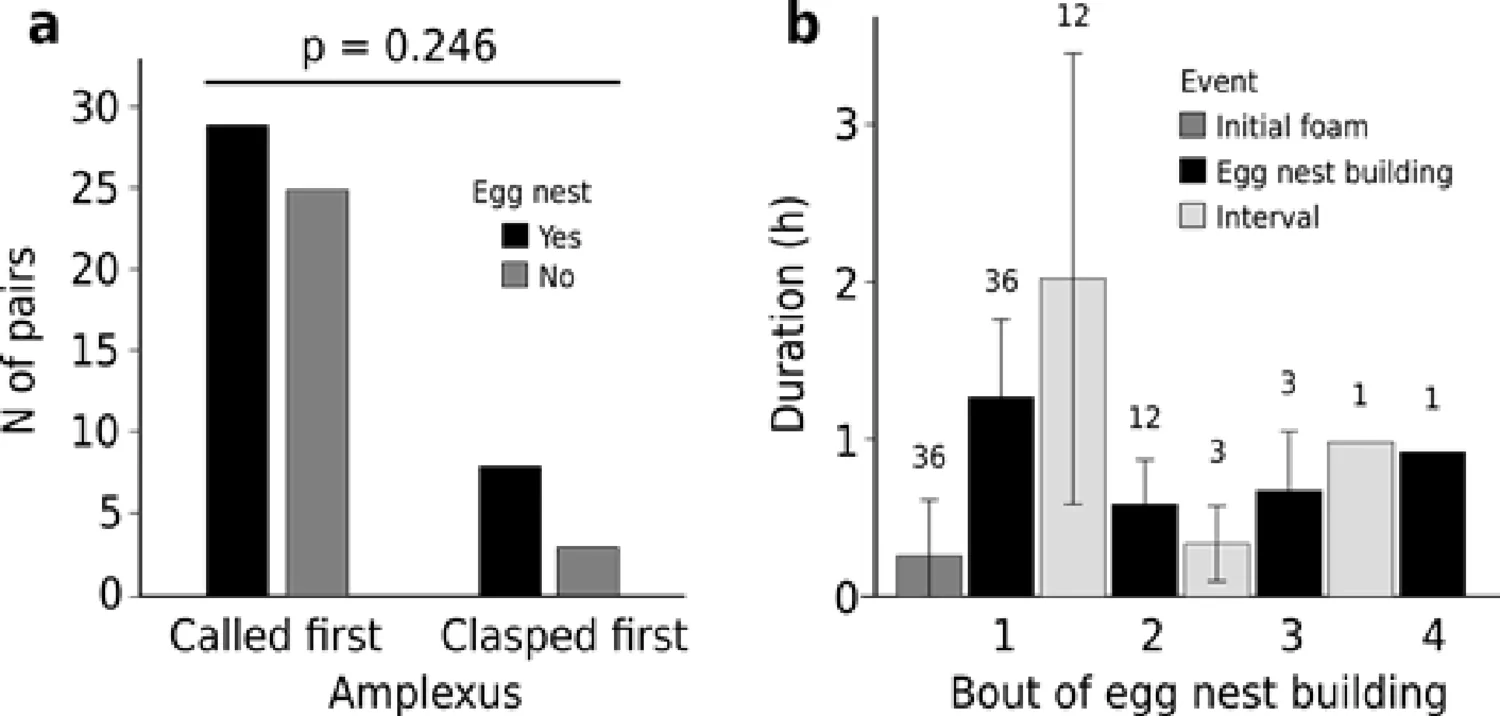
Egg nest building in the túngara frog. **a** Proportion of pairs that built an egg nest when the male had called first or clasped first. The proportions are not significantly different. **b** Duration of events in egg nest building. The number of cases of each group is indicated above the bar. Error bars = standard deviation

Of the 54 pairs in which the male called prior to amplexus, 54% produced an egg nest. Of the remaining 11 pairs in which the male did not call before amplexus 73% produced an egg nest. The difference in percent of egg nest building based on the occurrence of calling before amplexus was not significant however (*X*^*2*^ = 1.349, *P* = 0.246, *n* = 65; Fig. 7a). Most pairs built their foam nest in a single bout of activity that lasted 1.27 ± 0.49 h (*n* = 36; Fig. 7b). They produced a highly tridimensional floating structure composed of foam with small air bubbles that gave it an opaque white appearance which rendered the white eggs contained inside invisible. Some pairs preceded this activity with the production of a small amount of an initial foam that contained large bubbles, did not appear to contain eggs and tended to spread on the surface as opposed to growing vertically when the male whipped it. This initial foam production lasted from none to about 30 min and was followed by construction of the main nest but the distinction between these two phases was not obvious in some cases.

About 1/4 of the pairs produced a second bout of egg nest building that lasted 0.59 ± 0.28 h (*n* = 12) after an interval of 2.02 ± 1.43 h (Supplementary Materials S4_video_double_nest.mp4). During this interval, most pairs remained in amplexus but two of them separated, the male called, the female approached and he clasped her again. After the second bout of egg laying, three pairs remained in amplexus for an interval of about 20 min then added foam to the nest again. A single pair remained in amplexus for one more interval and resumed adding foam to the nest a fourth time for 0.92 h. The gradual growth of the nest volume was visible throughout each bout of nest construction. The end of this growth coincided with a change in posture of the male, and in most cases, a big change in position or orientation of the pair. It could also coincide with termination of amplexus. We observed a single case in which the pair started a new egg nest in a new location for their second bout of nest building. In all other cases, the pair returned to the edge of the nest and faced away from its center, resuming the original position to add more foam to the periphery of the nest. We disconsidered 3–4 cases in which the pair returned to the edge of the nest seemingly in egg laying position but the addition of new foam was not evident. When starting a new nest building bout, the frogs positioned themselves somewhat inserted in the original foam, so small additions or secretion of the initial foam would have been easily missed.

The results revealed that most males chose to call instead of clasping when at reaching distance from the female, that most males succeeded at clasping a female, that the female executed the last approach before amplexus, and that about half of the trials resulted in an egg nest. We encountered substantial variation in each of these outcomes however, and we asked if any of them involved differences in biometric variables or age of the male or of the female. If a male trait would differ between the ones that called first and the ones that clasped first, for example, this would indicate the potential existence of multiple male mating strategies determined by such traits. Similarly, if a female trait (ex: age) would differ between trials in which the male called first and the ones in which he clasped first, this would indicate that males could adjust their courtship to the trait value (age) of their mate.

The biometric data from our animals revealed that female SVL was highly correlated with body mass and more modestly wih BMI and age (all direct relationships; Table 1 left). The biometry of the males showed a high correlation between body mass and SVL or BMI but not with age (Table 1 right). When the influence of SVL, body mass, BMI, and age of the female and male were tested on the choice of the male to call first or clasp first considering all the trials, only male body mass was almost significant (*B* = 2.26, *P* = 0.052, *n* = 77; Supplementary Materials Fig. S5) indicating a potential for a modest tendency of heavier males being more likely to call before amplexus. When the effects of the same predicting variables were tested on the male’s success at clasping the female, no significant patterns were observed (Supplementary Materials Fig. S6).

**Table 1:** Pearson’s Correlation analysis of biometric data for females and males. Pearson Correlation (p-value)

	SVL	Female			SVL	Male		
		Body mass	BMI	Age		Body mass	BMI	Age
SVL	1	**0.781** **(< 0.001)**	**0.237 (0.039)**	**0.240 (0.037)**	1	**0.678 ** **(< 0.001)**	0.070 (0.546)	0.067 (0.564)
Body Mass		1	**0.785 (< 0.001)**	0.118 (0.307)		1	**0.773 (< 0.001)**	0.010 (0.929)
BMI			1	-0.041 (0.728)			1	-0.041 (0.727)
Age				1				1

N of each correlation is 76 or 77 and significant relationships are shown in bold

Among the 65 pairs that entered amplexus, we examined if SVL, body mass, BMI, and age of the female and male could predict which sex executed the last approach before amplexus. Among the predicting variables, we also included whether the male called first or clasped first. We found that only male BMI (*B* = -4.11, *P* = − 0.008) and calling or not first (*B* = 2.67, *P* = 0.011) had significant effects (Supplementary Materials Fig. S7). The last approach before amplexus was more likely to be executed by the male when he had a high BMI (good body condition) and he did not call before clasping the female. Using the same trials, we also asked if these same predicting variables could explain egg laying in our experiment. Only female BMI (*B* = 2.64, *P* = 0.020) was significant although female age was nearly significant (*B* = 0.004, *P* = 0.055 ; Supplementary Materials Fig. S8). Females were more likely to lay eggs when they had a higher BMI (likely more mature eggs). The raw data of this study are included in Supplementary Materials S9_raw_data.xlsx.

## Discussion

In a neutral arena removed from the chorus, the pair of túngara frogs bred with a similar timing and sequence of events as previously described in nature. Little variation could be attributed to age or body size. Multiple bouts of egg nest building were common, as was physical contact between the mates before and after amplexus. While neither animal had heard calls during the month prior to breeding, the presence and duration of calling varied among males and this did not predict the production of an egg nest by the pair.

### Timing

The timing of amplexus and egg laying in our trials was in agreement with those observed in Panama, where the lab colony was originated. Ryan (1983) described calling to start before sunset, peak ~ 3 h later and quickly subside 6 h after sunset. Calling in our trials also peaked 3 h after sunset and diminished greatly about 6 h after sunset but it increased again 9 h after sunset and then faded gradually until the end of the trial (Fig. 1). This late calling could be attributed to several causes. The continued presence of the female in close proximity may maintain the males stimulated to call. Alternatively, the males may have a higher energetic budget in our trials than in nature because they are free of parasites and possibly experience greater availability of food (Marler and Ryan 1996; Wilhite and Ryan 2024).

Mating and egg laying occurred throughout the night at Barro Colorado island, Panama in 1979, with a pronounced peak around 5.5 h after sunset (Ryan 1983, 1985). Some mated pairs left the water immediately after amplexus to only return hours later to lay eggs. Most of the breeding activity was observed between 5.5 and 7.5 after sunset but it quickly declined at later hours and little activity remained by 10 h after sunset. In our study, amplexus and egg laying also peaked 6–8 h after the artificial sunset and declined at later hours, although 12 h after the artificial sunset amplexus and nest building were still at about half of the peak levels. A potential explanation for the continued activity is that the sound isolation chambers did not contain a light source timed to produce a sunrise stimulus that could have summed up with the frogs circadian clocks to inhibit their breeding activity near sunrise time.

### Body size and age

Reproductive behaviors can vary in amphibians based on body size and age. (Dawson and Ryan 2009) reared túngara frogs in captivity and exposed them to silence, conspecific calls, or heterospecific calls during development to study the ontogeny of calling. They found that animals reared in silence produced shorter calls than the ones that listened to conspecific calls. They also found that juveniles exposed to heterospecific calls were later more likely to respond to heterospecific calls than juveniles exposed to conspecific calls (Dawson and Ryan 2012). This indicates that both the calls and the motivation to call can be modified by experience.

In adults, (Stănescu et al. 2022) explored age-related variability in *Leptodactylus bufonius* of Northern Argentina and found the fundamental frequency of the call to be related to size and age. In large aggregations, scramble competition can lead smaller males and those with inferior body condition to forgo calling and employ alternative mating strategies (Halliday and Tejedo 1995; Gastón and Vaira 2020). Studies on bullfrogs have found that satellite behaviors in the chorus were most commonly adopted by small, younger males (Howard 1978). Leary and collaborators (2005), however, examined choruses of toads (*Bufo cognatus* and *B. woodhousii*) and found that while satellite behaviors were mostly employed by small males, these tended to exhibit slower growth and were not younger. Males can therefore exhibit multiple mating strategies and the choice of strategy may be dependent on body size which can be influenced by age or physiological vigor.

Túngara frogs start calling with a body size of about 15–20 mm SVL while the average adult male size is near 30 mm (Guerra et al. 2014). The dominant frequency of the chuck component of the call, but not of the whine, tends to be correlated with body length and affect male mating success (Ryan 1985). This could, therefore, motivate a size-dependent choice of male mating strategy. We did not detect, however, differences in calling before amplexus, the last approach before amplexus, mating success, or egg laying that could be explained by age, body length, or body mass. This indicates that throughout the portion of ontogeny represented in this study (breeding adults), animals may follow the same general reproductive strategy. In the field, alternative breeding strategies have not been described in association with body size or age in the túngara frog. Mating success has been shown to be skewed toward large males but chorus attendance is not explained by body size (Ryan 1985).

Our data revealed that male body mass was highly correlated with BMI but not with age (Table 1). Among the males that clasped the female at least once, individuals that did not call first and had high BMI were more likely to execute the last approach before amplexus than those with low BMI (Supplementary Materials Fig. S7). On the other hand, when all active individuals were considered, we found a marginally significant effect of males with high body mass being more likely to call before amplexus (Supplementary Materials Fig. S5). The apparent conflict between these two results (larger males calling before amplexus or not) occurred because males that did not call formed two groups and one of them was excluded from the analysis of approach to amplexus. In the body mass test, males with small body mass called and clasped the female but she never laid eggs. These males were not included in the approach test, in which males with higher BMI (and larger) clasped without calling and executed the last approach before amplexus. Success at egg laying was not explained by male traits, however, but by female BMI (Supplementary Materials Fig. S8). High BMI for females should be a reflection of them bearing more mature eggs. Both female BMI and age had a mild but significant direct correlation with SVL but body length did not explain success at egg laying.

### Egg nest building and multiple bouts

Egg nest building in túngara frogs is a coordinated activity in which the female expels the eggs embedded in a gel that the male can whip into a foam using cyclic movements of its hind limbs (Heyer and Rand 1977). The gel contains 6 major proteins, one of them with a surfactant activity that promotes the formation of foam (Fleming et al. 2009). Production of the egg nest takes slightly more than one hour (Ryan 1985; Dalgetty and Kennedy 2010) and it is highly energetically demanding (Bucher et al. 1982). Construction of the nest involves 3 phases: a short initial phase in which the female lays no eggs and the male builds a bubble raft with her secretions; a long nest building phase during which the eggs are laid; and a very brief termination phase (Dalgetty and Kennedy 2010). Multiple bouts of egg laying have not been reported to occur spontaneously in the field. Dalgetty and Kennedy (2010), however, noted that both in the lab and in the field, pairs that are disturbed during nest building tend to return to their original positions and continue to add foam to the nest. In addition, communal foam nests have been documented involving multiple pairs (Ryan 1985; Dalgetty and Kennedy 2010). The resulting nests have a reduced area to volume ratio which can confer enhanced protection to the developing eggs against evaporative water loss if the pond dries out (Zina 2006).

Most pairs in the lab built egg nests in a single bout and they did so in 1.27 h on average. Our observations on initial foam production match, however, the description by Dalgetty and Kennedy (2010) for the initial phase of nest construction. With the production of the initial foam included the nest building time increases to 1.5 h indicating that the animals in our experiment may have built more slowly than the ones in the field (Ryan 1985; Dalgetty and Kennedy 2010). We did not detect pauses in nest volume increase or pauses in the movements of the male during a bout of nest building that were long enough to be salient with our low time resolution imaging. The lab animals may therefore have worked at a slower pace as opposed to having taken pauses.

The common occurrence of multiple amplexuses and egg laying in the lab with no reports in the field could have multiple explanations. We speculated that it could have been caused by lack of stimulation of the females due to the absence of a chorus. If a single male calling did not suffice to excite the female or produced slow ovulation maybe this caused her to not have all her mature eggs ready to lay in the first bout of nest building. This could potentially also occur in the field during nights in which few males are calling. As a non-exclusive alternative, the observation by Dalgetty and Kennedy (2010) of nesting pairs being disturbed demonstrates that they can return to resume egg nest building in the field.

### Non-amplexus contact and last approach before amplexus

Physical contact that did not immediately lead to amplexus was common in our study and could occur before or after amplexus. In addition, females were more likely than males to execute the last approach before amplexus. Close-range courtship in frogs involves a variety of behaviors. The female has been reported to approach the male and physically touch him immediately before amplexus in various groups (Martof and Thompson 1958; Barreto and Andrade 1995; Chuang et al. 2013). Some females emit short calls during courtship (Emerson 1992; Bush 1997; Bosch and Márquez 2001; see review in Toledo et al. 2015). In the murky waters of African ponds, visibility is low and frogs that live underwater as adults employ subaquatic acoustic signals to attract mates and perform duetting during courtship (Österdahl and Olsson 1963; Tobias et al. 1998; Elepfandt et al. 2022).

Within the family of the túngara frog (Leptodactylidae) the female in some species is escorted by the male to a burrow and may make physical contact prior to amplexus, such as in *Leptodactylus mystacinus* (Oliveira Filho and Giaretta 2008) and *L. fuscus* (Lucas et al. 2008). In *L. bufonius* the pair may exchange calls and vibratory lateral movements of the body before entering a burrow to lay eggs (Faggioni et al. 2017) and this reciprocal calling may have also been observed in *L. fragillis* (Bernal and Ron 2004). In the subfamily Leiuperinae, females of *Physalaemus cuvieri* (Barreto and Andrade 1995) and *P. ephipifer* (Hödl 1990) have been reported to make a single physical contact with the male shortly before initiating amplexus (Barreto and Andrade 1995; Brasileiro and Martins 2006; Cajade et al. 2020). This behavior has not been observed, however, in *P. santafecinus*,* P. albonotatus* (Cajade et al. 2020) or *P. centralis* (Brasileiro and Martins 2006). Physical contact before amplexus has therefore been documented in the túngara frog subfamily. In túngara frogs, Ryan (1983) reported that the female commonly approaches and contacts the male immediately before he clasps her. Physical contact was also reported from experiments in a 184 × 92 cm seminatural arena (Akre and Ryan 2011). The authors reported various behavioral patterns of the female preceding amplexus and counted the number of occurrences of a “body bump” contact with the male. They also reported that females, when visible to the male, often produced displacements that did not bring them closer to or away from the male but stimulated the male to call more vigorously. This behavior may help females to select the most vigorous males but it may also allow females to elicit the level of stimulation necessary for egg laying. Some of the non-amplexus contact observed in our study could have a similar role. These interactions might be suppressed within large choruses in the context of social stimulation extrinsic to the pair.

We inferred females to be almost 4 times more likely than males to produce the last approach before amplexus. Field studies have reported calling males to be mostly stationary but clasp other males that approached them within 5–10 cm (Ryan 1985). The female was reported to listen to males from up to 10–20 cm distance and then move directly toward the elected mate which would clasp her. In our lab trials, however, females frequently moved next to the male and even touched him without being clasped. This indicates that close proximity may not always be the trigger for initiation of amplexus and other interactions might be involved which we may have missed due to the low time-resolution of our setup. As an alternative explanation, the lack of competition by other males may allow the male in the lab to avoid the risk of attempting amplexus too early and instead wait for the female to demonstrate her receptivity from a closer distance.

### Calling

When males were released into the breeding enclosures, they encountered water and a female at reaching distance. Some males clasped the female while the majority entered the water and called before attempting amplexus. This variability may be explained at multiple levels (Tinbergen 1963), see English translation (Tinbergen 2005). At the ultimate level, male calling may arouse the female and increase his chances of clasping her or building an egg nest with the female, as discussed previously. At the proximate level, one may ask about the neural and endocrine structures that govern the response of the male to the available stimuli (water to call from, female) and the potential effect of lack of stimulation by the chorus.

The male frog’s motivation to clasp the female has been shown to depend on testosterone (Moore et al. 2005). In *Xenopus laevis*, Kelley and Pfaff (1976) found that castration abolished amplexus but it could be recovered with a testosterone implant. Ishii and Itoh (1992) studied *Bufo japonicus* and provided evidence of inverted cause and effect with amplexus causing an increase in plasma sex-hormone levels. This was verified in *Bufo marinus* which showed increased androgen levels but not corticoid level when clasping females (Orchinik et al. 1988). In a well controlled experiment, Freiler and colleagues (2026) showed that participation in amplexus increases the circulating levels of testosterone and estradiol but not corticosterone in *Hyla chrysoscelis.* They also showed that concentrations of these same hormones can predict the calling performance of the male (Baugh et al. 2026). Studies in salamanders have shown that the hormone arginine vasotocin (AVT), a homologue of the mammalian vasopressin, can also stimulate amplexus when injected into males while injection of vasotocin antagonists reduced the incidence of amplexus (Moore and Miller 1983). Vasotocin regulates various aspects of social behavior in non-mammal vertebrates (Wilczynski et al. 2017). In *Taricha granulosa*, AVT was shown to influence olfactory and visual sensorimotor processes specific to sexual attraction to the female (Thompson and Moore 2000). The mechanism that mediates a male’s choice between calling and clasping or that leads him to interrupt calling to clasp the female is not yet clear. It could involve a response to a change in hormone levels but it could also be a neural response to a sensory cue (Moore et al. 2005).

The relevance of behavioral observations obtained in small choruses or in the lab may appear questionable for not fitting the context that is experienced by most individuals at the chorus. These observations can be useful tools, however, in the identification of the behavioral and physiological mechanisms that control mating behavior. The prompt mobilization of the males in our neutral arena, in spite of lack of chorus sounds, was remarkable with most animals mating within a few hours. It indicates that stimulation by water or the presence of the female is sufficient to excite the neuroendocrine system of the male and, via courtship, of the female to the levels necessary for reproduction. Conversely, the occurrence of multiple amplexuses or multiple egg laying in some pairs may indicate that reproduction could have occurred near the minimum required levels of arousal in the pair. Each of these aspects can be further tested and advance our understanding of breeding behavior in ways that are complementary to the wonderful complexity of natural systems that is unveiled by field studies.

## Conclusion

In a breeding setting without social stimulation extrinsic to the pair, túngara frog males may call or not before clasping the female. Reproduction may be partitioned into multiple amplexuses or egg nest construction bouts and the pair exhibits an abundance of non-amplexus contacts that have only been observed in small choruses. Explaining these patterns will require elucidating the responses of the animals to individual stimuli within the pair and extrinsic to it. The framework of the current experiment may prove useful in this endeavor which can help elucidate the mating behavior of this emerging model organism.

This study characterized novel aspects of the breeding biology of the túngara frog through close examination of the interactions within a pair during courtship. This was made possible by methodological developments that enabled controlled breeding and close-up video monitoring of this secretive behavior. This achievement was inspired by Peter Narins’ rich legacy of exploration and innovation (Narins and Capranica 1978; Lewis and Narins 1985). The studies he conducted or guided show a variety of ways in which sensitive equipment can be adjusted to operate in remote places ranging from humid tropical forests to the driest deserts and reveal novel biological mechanisms (Narins et al. 1997, 2003, 2005; Arch et al. 2009; Narins and Meenderink 2014). These studies also demonstrate a motivation to cross the world after the ideal species to answer a physiological question with, study it in the field, and then bring it back to the lab environment when more controlled data collection is required (Willi et al. 2006a, 2026b; Gridi-Papp et al. 2008; Farahbakhsh and Narins 2008; Arch et al. 2009). This example of versatility and unhindered pursuit of answers through scientific exploration and innovation is an asset for present and future scientists in experimental biology.

## Supplementary Information

Below is the link to the electronic supplementary material.

Supplementary Material 1 S2 is A picture panel showing an example of 4 non-amplexus contacts within a single trial. In each case, the female placed the palm of one or both forelimbs on the male’s back

Supplementary Material 2 S5-8 are four panel figures with bar plots showing comparisons of age and body size between females and males and their effects on male calling or clasping, chances of amplexus, sex that executes the last approach before amplexus, and chances of building an egg nest

Supplementary Material 3 Spreadsheet containing the biometric and behavioral data analyzed in this study

Supplementary Material 4 Time-lapse video file showing 24 h of monitoring in 2 min. It includes the entire trial for a pair with male calling, female approach and non-amplexus interactions, amplexus, egg nest building and subsequent calling. The female is already in the container when the male is added, but she is initially at a high position on the wall where she is out of the field of view of the camera. Once the males starts calling, she comes down and starts interacting with him. Notice the real time displayed on the top left

Supplementary Material 5 Time-lapse video file showing the male calling at the base of the ramp while the female descends the entire ramp moving backwards until she meets the male and he clasps her. Notice the real time displayed on the top left

Supplementary Material 6 Time-lapse video file showing an egg nest being built in two bouts. Notice the real time displayed on the top left

## Data Availability

All data supporting the findings of this study are available within the paper and its Supplementary Information.

## Declarations

### Research involving animals

This study followed IACUC protocol 22R07 of the University of the Pacific.
