# Supplementary material for: Courtship at reaching-range in the túngara frog
Source: J Comp Physiol A Neuroethol Sens Neural Behav Physiol. 2026 Aug 10;212(5):765–79. doi: 10.1007/s00359-026-01830-4 (PMC13642479; doi:10.1007/s00359-026-01830-4)
Supplement: Supplementary file 1 [file 359_2026_1830_MOESM1_ESM.pdf]

Supplementary Materials

Courtship at reaching-range in the túngara frog

Journal of Comparative Physiology A

Olivia R. Chung and Marcos Gridi-Papp

Corresponding author: Marcos Gridi-Papp. Department of Biological Sciences, University of the Pacific; Stockton, 95211, USA.

Email: mgridipapp@pacific.edu

This file includes:

Figure S2 with captions.

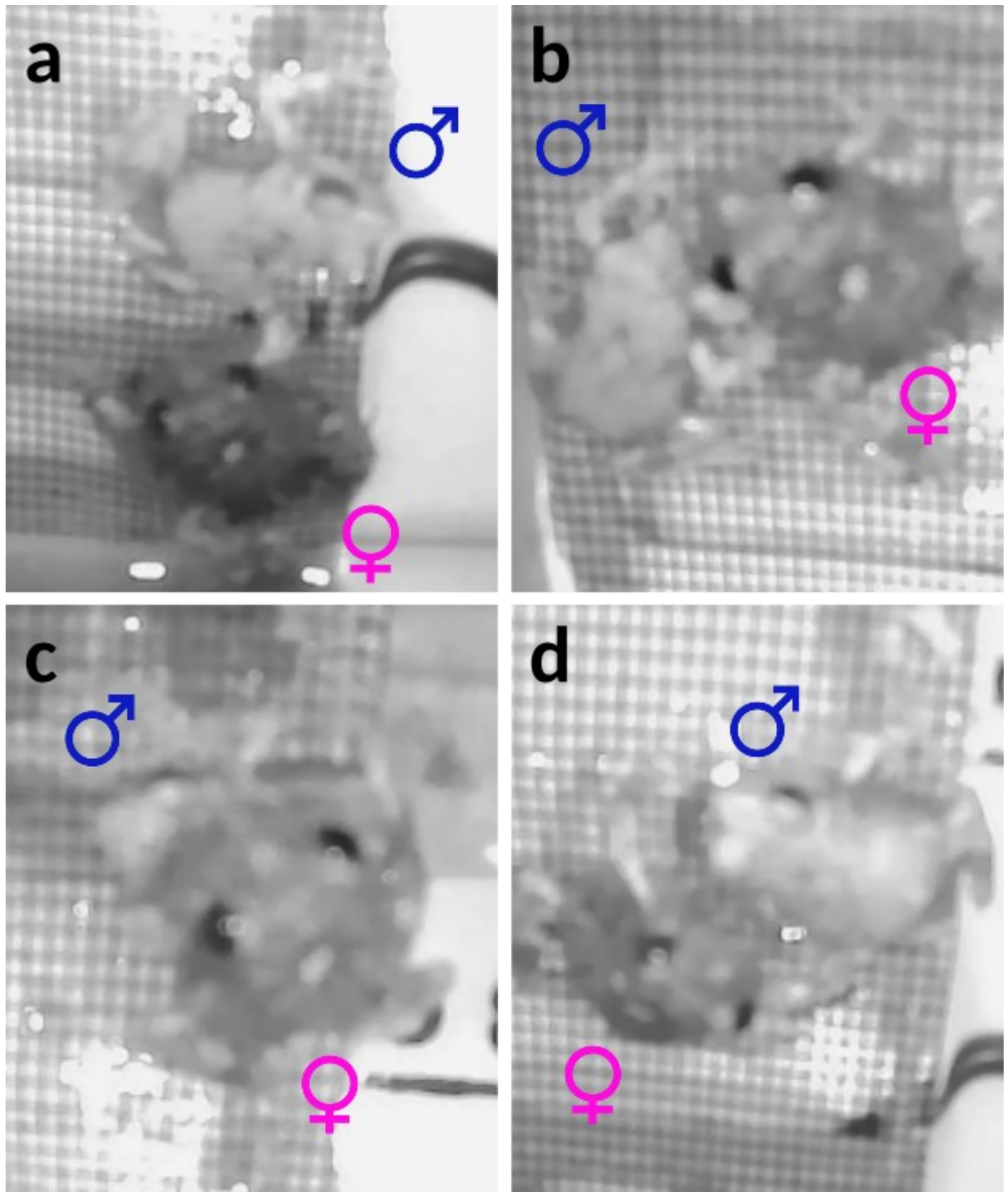

Fig. S2 Physical contact not immediately followed by amplexus in the túngara frog. The male (smaller, lighter colored) refrained from calling and explored the enclosure but spent most time on the dry portion of the ramp. The female (larger, darker colored) was highly vagile exploring the enclosure and approached the male at multiple times on the ramp, touching his back with the palm of a forelimb. **a** Contact at 6:20 h. **b** Contact at 6:37 h. **c** Contact at 7:15 h. **d** Contact at 8:36 h. All times relative to the artificial sunset time in the frog colony. All contacts occurred in a same trial night. Images obtained with infrared illumination.
