# Supplementary material for: Courtship at reaching-range in the túngara frog
Source: J Comp Physiol A Neuroethol Sens Neural Behav Physiol. 2026 Aug 10;212(5):765–79. doi: 10.1007/s00359-026-01830-4 (PMC13642479; doi:10.1007/s00359-026-01830-4)
Supplement: Supplementary file 2 [file 359_2026_1830_MOESM2_ESM.pdf]

Supplementary Materials

Courtship at reaching-range in the túngara frog

Journal of Comparative Physiology A

Olivia R. Chung and Marcos Gridi-Papp

Corresponding author: Marcos Gridi-Papp. Department of Biological Sciences, University of the Pacific; Stockton, 95211, USA.

Email: mgridipapp@pacific.edu

This file includes:

Figures S5, S6, S7 and S8 with captions.

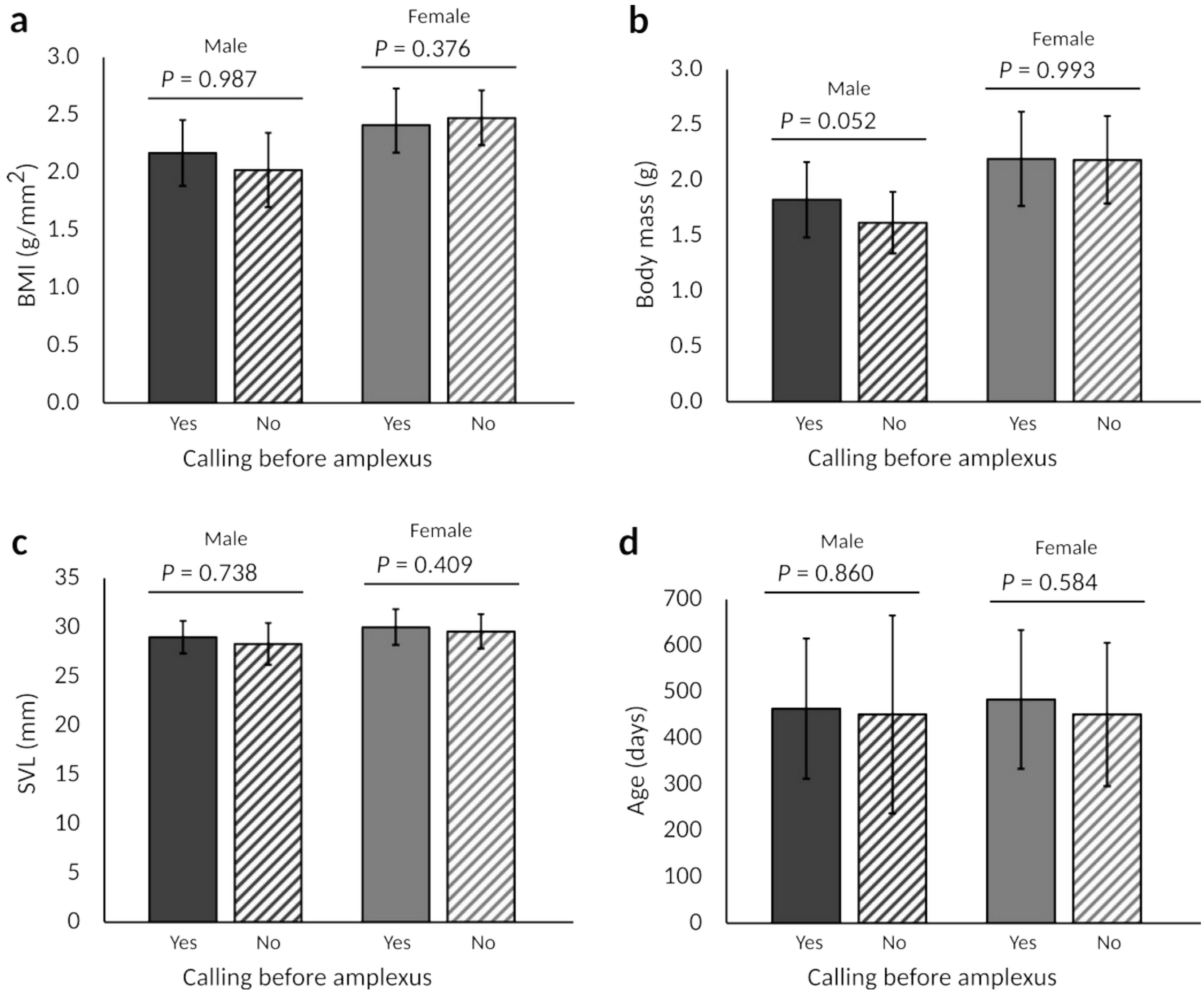

Fig. S5 Male and female biometry and its ability to predict the male choice of calling or not before amplexus, analyzed with a logistic regression. **a** Body mass index (BMI) differences by sex. **b** Body mass differences by sex. **c** Snout to vent length (SVL) differences by sex. **d** Age differences by sex. Males that called first had larger body mass (b) than males that clasped first. P-values above the bars indicate significance as a predictor in the logistic regression model. Error bars show standard deviation

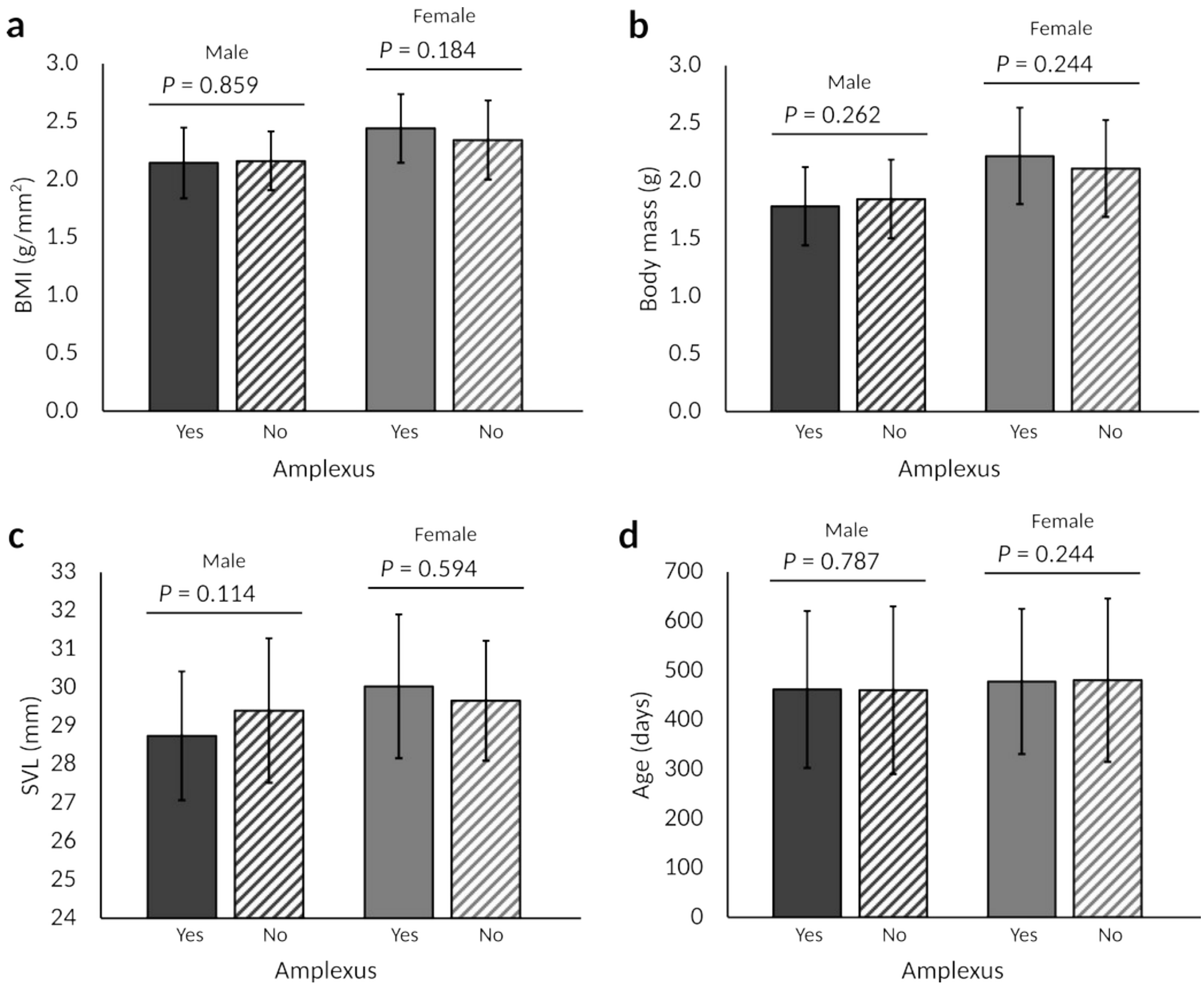

Fig. S6 Male and female biometry and its ability to predict the occurrence of amplexus, analyzed with a logistic regression. **a** Body mass index (BMI) differences by sex. **b** Body mass differences by sex. **c** Snout to vent length (SVL) differences by sex. **d** Age differences by sex. Male success at clasping a female was not predicted significantly by any of the biometric variables. P-values above the bars indicate significance as a predictor in the logistic regression model. Error bars show standard deviation

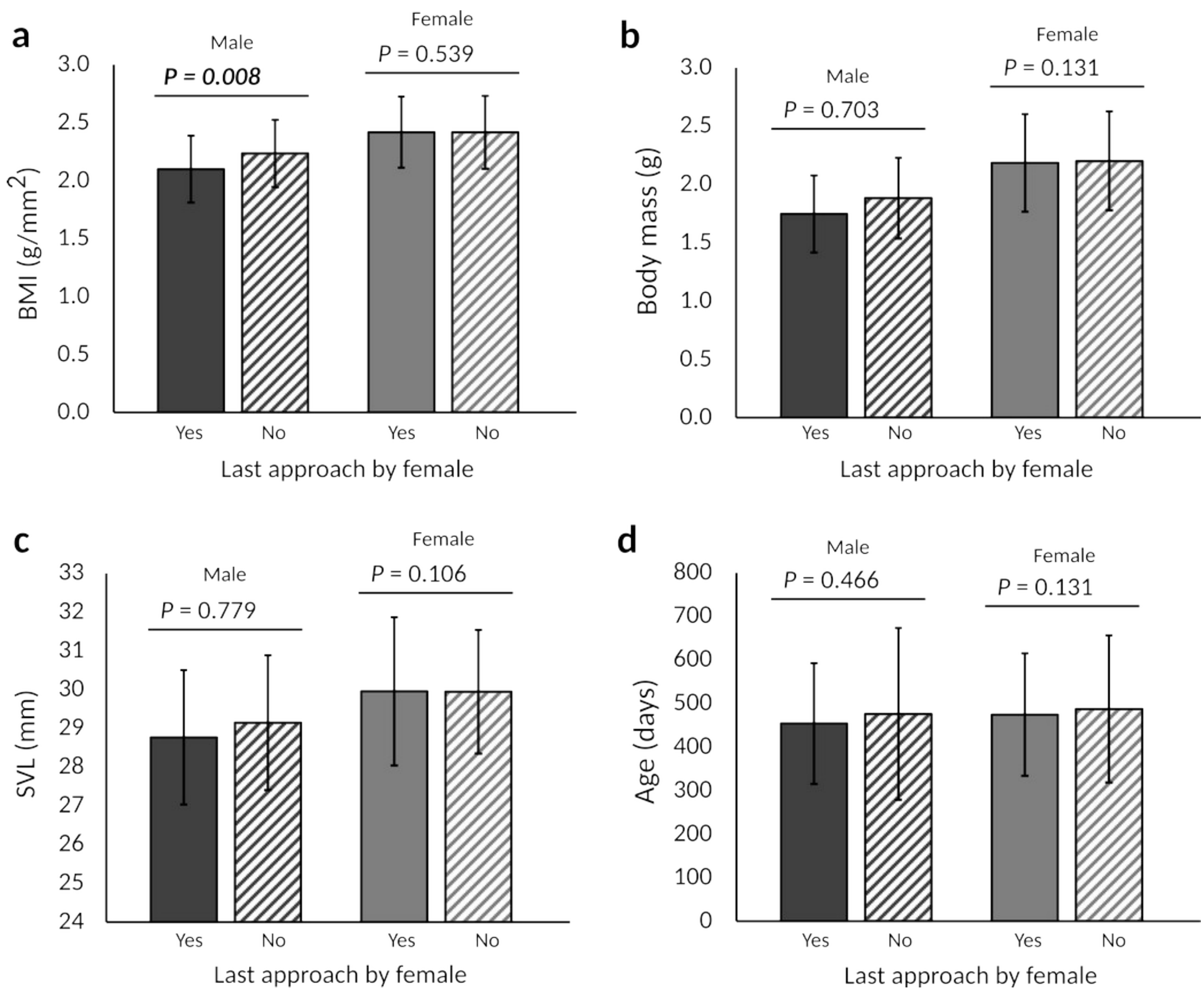

Fig. S7 Male and female biometry and its ability to predict who executes the last approach before amplexus, analyzed with a logistic regression. **a** Body mass index (BMI) differences by sex. **b** Body mass differences by sex. **c** Snout to vent length (SVL) differences by sex. **d** Age differences by sex. Males with higher BMI (a) tended to execute the last approach before amplexus. P-values above the bars indicate significance as a predictor in the logistic regression model. Error bars show standard deviation

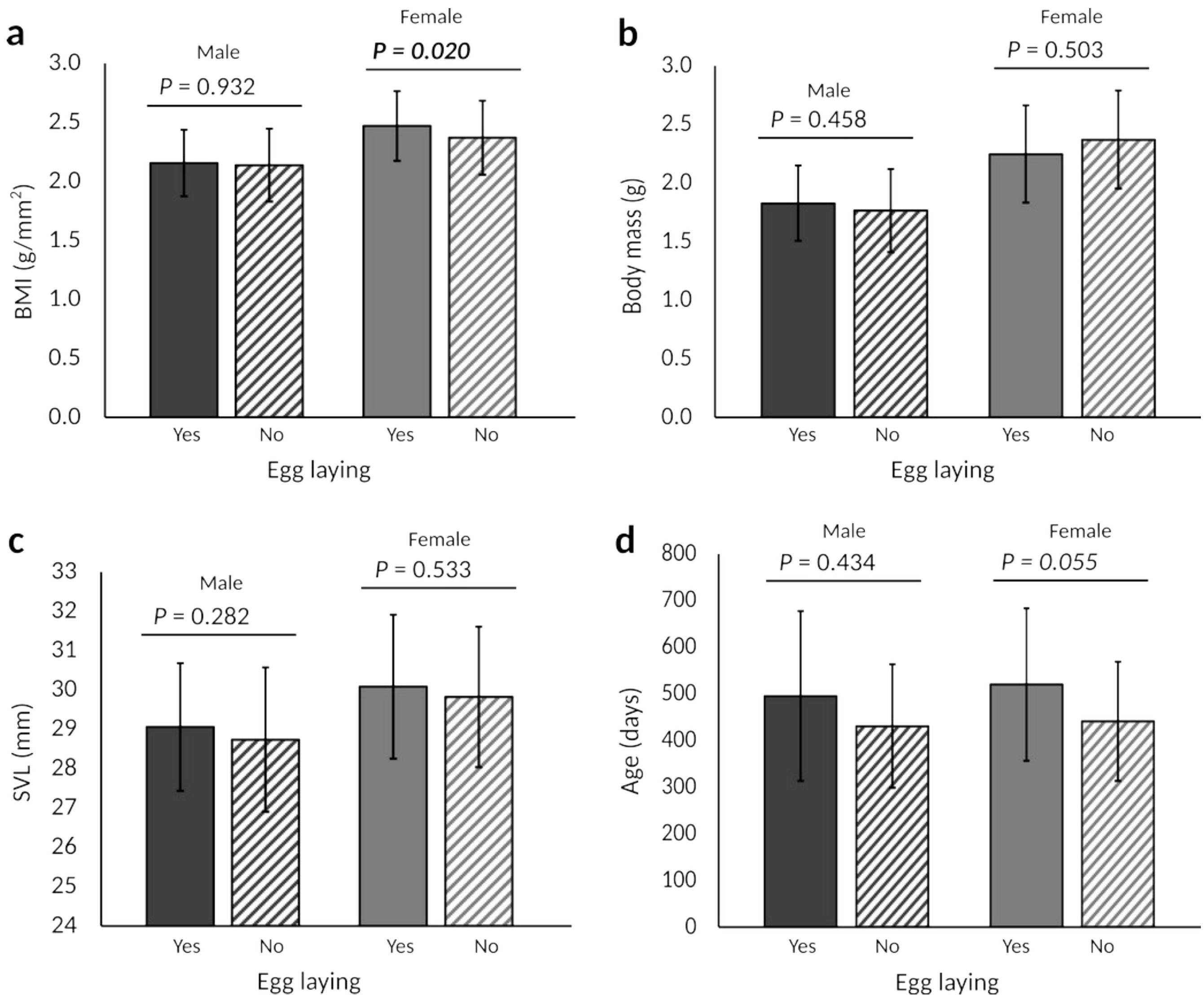

Fig. S8 Male and female biometry and its ability to predict the production of an egg nest, analyzed iwit a logistic regression. **a** Body mass index (BMI) differences by sex. **b** Body mass differences by sex. **c** Snout to vent length (SVL) differences by sex. **d** Age differences by sex. Females with a higher BMI (a) and older (d) were more likely to produce an egg nest. P-values above the bars indicate significance as a predictor in the logistic regression model. Error bars show standard deviation
